# Social Cognition in Children With Non-specific Intellectual Disabilities: An Exploratory Study

**DOI:** 10.3389/fpsyg.2020.01884

**Published:** 2020-08-11

**Authors:** Emilie Jacobs, Poline Simon, Nathalie Nader-Grosbois

**Affiliations:** UCLouvain, Psychological Sciences Research Institute, Louvain-la-Neuve, Belgium

**Keywords:** social cognition, theory of mind, social information processing, intellectual disability, social behavior

## Abstract

Social cognitive abilities – notably, Theory of Mind (ToM) and social information processing (SIP) – are key skills for the development of social competence and adjustment. By understanding affective and cognitive mental states and processing social information correctly, children will be able to enact prosocial behaviors, to interact with peers and adults adaptively, and to be socially included. As social adjustment and inclusion are major issues for children with intellectual disabilities (IDs), the present study aimed to explore their social cognitive profile by combining cluster analysis of both ToM and SIP competence, and to investigate the structure of relations between these skills in children with IDs. Seventy-eight elementary school children with non-specific IDs were recruited. They had a chronological age ranging from 4 years and 8 months to 12 years and 6 months and presented a preschool developmental age. Performance-based measures were administered to assess ToM and SIP abilities. Questionnaires were completed by the children’s parents to evaluate the children’s social competence and adjustment and their risk of developing externalizing or internalizing behaviors. Exploratory analysis highlighted strengths and weaknesses in the social cognitive profiles of these children with IDs. It also emphasized that the understanding of affective and cognitive mental states was used differently when facing appropriate vs. inappropriate social behaviors. The present study leads to a better understanding of the socio-emotional profile of children with IDs and offers some suggestions on how to implement effective interventions.

## Introduction

In typically developing children, theoretical conceptions and developmental studies emphasize that the preschool period, ranging from 3 to 6 years, corresponds to a “critical” period of development of emotional and social abilities (Vygotsky, 1978; Perner, 1991; Wellman, 1991; Wellman et al., 2001; Wellman and Liu, 2004; Astington and Baird, 2005; Astington and Edward, 2010). The diversity of social interactions with peers and adults in different contexts increases, and social environments require more and more respect for social conventions or rules. Children have to develop new abilities to understand emotional and social situations and to interact successfully with others, in order to be perceived as socially adjusted and to create harmonious social relationships. However, children with intellectual disabilities (IDs) find it difficult to acquire these emotional and social abilities. In the definition of intellectual disability, limitations in both cognitive and adaptive functioning, including social skills (Schalock et al., 2010; American Psychiatric Association, 2013), are recognized as diagnostic criteria. To navigate the world, children with IDs have to develop skills in social cognition to be able to interact in a socially appropriate and adaptive way (Yeates et al., 2007). However, a majority of children with IDs face difficulties in social cognition and perform at a lower level in comparison with typically developing children with the same chronological or developmental age (Leffert and Siperstein, 2002). Depending on whether subjects are matched for developmental or chronological age, studies have shown that there is either a deficit or a delay in social cognitive development in children with IDs, in comparison with typically developing children. These comparisons determine whether impairments are related to a child’s specific disorder or to developmental difficulties (Skwerer, 2017). Social cognition in children with IDs has been explored according to either a developmental approach through the concept of Theory of Mind (ToM) or a functional view based on the social information processing (SIP) model. By contrast with previous studies investigating specific aspects of either ToM or SIP, our study examined social cognitive profiles according to both approaches.

### ToM in Children With IDs

ToM is described as the ability to understand one’s own and other people’s mental states, and to infer other people’s mental states in order to predict social behavior and to behave in a socially adapted way (Denham et al., 2003; Barisnikov et al., 2002; Deneault and Ricard, 2013). From a Vygotskyan perspective, ToM development is related to language acquisition and social interactions in the family, social and cultural environment (Ricard et al., 1999). Children with IDs face difficulties in both these areas but also in developing early prerequisites of ToM abilities (such as imitation, pretend play or joint attention; Charman et al., 2000; Tourrette et al., 2000; Meltzoff, 2002; Rakoczy, 2008; Barthélémy and Tartas, 2009) and empathy (Sigman et al., 1992). The distinction between affective and cognitive ToM is relevant here, as the nature of the mental states being considered makes a difference to whether a delay or a deficit is reported (Deneault and Ricard, 2013). Affective mental states include desires and emotions, while cognitive ones include beliefs, pretense, visual perception, intentions, false beliefs, knowledge, thinking, and attention (Flavell, 1999). In terms of affective ToM, children with IDs present a delay in their understanding of causes and consequences of emotions (Garitte, 2003; Thirion-Marissiaux and Nader-Grosbois, 2008b; Fiasse and Nader-Grosbois, 2012; Baurain and Nader-Grosbois, 2013), when compared with typically developing children matched for developmental age. In terms of cognitive ToM, results of past studies have suggested the existence either of a delay (Giaouri et al., 2010; Fiasse and Nader-Grosbois, 2012) or of a deficit (Charman and Campbell, 2002; Thirion-Marissiaux and Nader-Grosbois, 2008a), depending on the tasks (e.g., unexpected content task, appearance-reality task, change of location task), thus revealing an intertask variability. More particularly, perspective-taking competence seems deficient in children with borderline IDs, who struggle with distancing themselves from their own perspective to understand what others know (Baglio et al., 2016). Finally, Légaré et al. (2019) observed that mothers also perceived difficulties in both affective and cognitive ToM competence in their children with IDs, compared with parents of typically developing children.

ToM abilities lead to better social interaction and adjustment (Charman and Campbell, 1996, 2002; Barisnikov et al., 2002; Abbeduto and Murphy, 2004; Jervis and Baker, 2004; Deneault and Ricard, 2013). More particularly, some specific and variable links have been emphasized between, on the one hand, affective and cognitive ToM abilities in children with IDs, and on the other hand, their social or prosocial behavior during interactions with peers and adults as perceived by teachers (Fiasse and Nader-Grosbois, 2012; Thirion-Marissiaux and Nader-Grosbois, 2008c) or observed in dyadic play (Baurain and Nader-Grosbois, 2013).

### SIP in Children With IDs

The SIP model was conceived to explain the cognitive processes that contribute to the understanding and resolving of social situations in typically developing children and children presenting externalized behavior disorders (Crick and Dodge, 1994). Individuals treat social information according to specific steps – (1) the encoding of emotional and social cues, (2) the interpretation of these cues, (3) goal clarification, (4) the generation of possible responses, and (5) response selection – displayed in social situations (Crick and Dodge, 1994). Problems in one or more steps will lead to a risk of maladjusted social behavior. More recently, this SIP model has been applied in studies of children with IDs (van Nieuwenhuijzen et al., 2006). This functional approach has improved our understanding of impairments in social problem-solving skills in children with IDs (Baurain and Nader-Grosbois, 2013; Wieland et al., 2014), by identifying the SIP steps in which the deficiencies lie. Some studies have shown that children with IDs display difficulties in encoding (step 1) and interpreting social and emotional cues (step 2) (van Nieuwenhuijzen et al., 2004; van Nieuwenhuijzen et al., 2009). These difficulties are particularly observed in socially ambiguous or provoking situations, in which these children are more likely to have faulty detection of information and to misidentify cues indicating unintentional actions (Jahoda et al., 2006; van Nieuwenhuijzen and Vriens, 2012). In situations where negative cues occur, hostile attribution bias is more likely to occur in children with IDs (Leffert et al., 2010), whereas once it is clear that there are no hostile intentions, children display no difficulty (Crick and Dodge, 1994; Leffert et al., 2000). Problems in encoding and interpretation result in deficits in steps 4 and 5 (Leffert and Siperstein, 1996; van Nieuwenhuijzen et al., 2009; van Nieuwenhuijzen and Vriens, 2012), such as positive evaluation of maladjusted behavior and production of aggressive reactions. Difficulties in social competence and adjustment, such as behavioral problems (Leffert and Siperstein, 1996; van Nieuwenhuijzen et al., 2004, 2009; van Nieuwenhuijzen and Vriens, 2012; Baurain and Nader-Grosbois, 2013), could therefore be explained by the specific SIP profile of these children.

### Objectives of the Present Study

No previous study has examined the social cognition of children with IDs by combining analysis of both ToM and SIP profiles in order to better understand how their particular profiles contribute to their social adjustment or to the risk of maladjustment in family or school contexts. Studies have reported that these children are more at risk of displaying externalizing behaviors (such as opposition, resistance or aggressiveness; Taylor, 2002; Rojahn et al., 2012) and internalizing behaviors (such as withdrawal, isolation or anxiety; Merrell and Holland, 1997; Thirion-Marissiaux and Nader-Grosbois, 2008c), or even both kinds of behavioral problems (Dekker et al., 2002; Baker et al., 2003; Dekker and Koot, 2003; Emerson, 2003; Nader-Grosbois et al., 2013; Hauser-Cram and Woodman, 2016; Bailey et al., 2019). To make it possible to give effective support to children with IDs in developing their social abilities and gaining social inclusion, the strengths and weaknesses in their social cognitive profiles, particularly in affective and cognitive ToM as well as in SIP, need to be explored. The present study aimed firstly to identify clinical homogenous groups of children depending on their affective and cognitive ToM abilities and/or SIP competence. Cluster analyses were applied considering children’s social cognitive skills and between-group comparisons were realized regarding their individuals’ characteristics and social behaviors. We hypothesized that children with IDs belonging to a cluster described by better ToM and SIP abilities should have higher developmental age and better social emotional and behavioral competence, in comparison with a cluster presenting less social cognitive abilities. The second objective was to analyze the structure of relations between skills related to affective and cognitive ToM and SIP processes in positive or negative social situations in children with IDs. We hypothesized that ToM abilities and SIP skills would be related depending on the situation. Social situations required ToM abilities such as perspective taking, comprehension of beliefs or emotions, and SIP skills needed to process social information adequately and to solve social problems.

## Materials and Methods

### Participants

Seventy-eight children (56 boys and 21 girls) with non-specific IDs were recruited in special primary schools from French-speaking areas of Belgium. They had been diagnosed as having mild to moderate IDs (intelligence quotient between 50 and 70), according to AAIDD (American Association on Intellectual and Developmental Disabilitites, 2011) and DSM-V (the *Diagnostic and Statistical Manual of Mental Disorders*) criteria. The intelligence quotient was not assessed by the experimenter but was checked through a cognitive assessment made previously by a professional. Children had to present an intelligence quotient between 50 and 70 to meet inclusion criteria. As can be seen in Table 1, the children were approximatively 9 years, with a mean chronological age of 109.86 months (*SD* = 21.19), ranging from 56 to 150 months. Their global developmental age was about 5 years, with a mean of 63.97 months (*SD* = 13.65), ranging from 40 and 91 months. Moreover, their estimated verbal developmental age was between 37 and 86 months (mean = 62.88; *SD* = 13.71). Before we started the recruitment, an ethics committee of the faculty of psychology at UCLouvain approved the research procedure, notably by attesting to the respect of the ethical guidelines of the declaration of Helsinki. Recruitment was restricted on the basis of exclusion criteria. Children with Williams’s syndrome or autistic spectrum disorder could not be included. Children also had to be able to form sentences of three to four words and display a global developmental age higher than 36 months. Before connecting with parents, we asked school directors to indicate children who potentially met these criteria. Despite this and due to the application of these strict criteria, six children were excluded. Parents received a consent form from teachers explaining the research goal and procedure. This consent form also offered the possibility to receive, at the end of the procedure, a report of the child’s competence based on the completion of the different measures. The children’s families presented a low socioeconomic status. On a nine-level scale describing range of monthly income from 0–500 to 4,000 and more, the parents reported a low income (mean = 3.14) corresponding to a monthly income (salaries and benefits) of 1,000–1,500 euros, compared to a mean monthly salary of about 1,527 euros in Belgium. In terms of the parents’ levels of education, the mothers had typically completed secondary school (mean = 3.24), while the fathers had typically completed an apprenticeship contract (mean = 4). Our sample therefore revealed a certain homogeneity of cultural and socioeconomic status.

**TABLE 1:** Demographic and individual characteristics: mean scores and standard deviations in Theory of Mind, Social information processing, and social (mal)adjustment measures.

Variables		Mean
**Children with non-specific IDs (*n* = 78)**		
Sex (% boys)		73%
CA (in months)		109.86 (21.2)
GDA (in months)		63.97 (13.65)
VDA (in months)		62.88 (13.71)
Family measures	Family income	3.14 (1.06)
	Mothers’ education (max = 7)	3.24 (2.25)
	Fathers’ education (max = 7)	4 (1.72)
Explicit ToM measures	ToM Task Battery total (max = 15)	8.14 (2.37)
	Affective ToM Task Battery (max = 6)	5.04 (1.11)
	Cognitive ToM Task Battery (max = 6)	2.58 (1.45)
	Mixed ToM Task Battery (max = 3)	0.63 (0.91)
	ToM emotions (max = 12)	7.62 (2.33)
	ToM emotions – causes (max = 6)	4.07 (1.46)
	ToM emotions – consequences (max = 6)	4.21 (1.78)
	ToM beliefs (max = 5)	2.9 (1.32)
Problem-solving task	Judgment score on appropriate vignettes (max = 2)	1.71 (0.49)
	Judgment score on inappropriate vignettes (max = 2)	1.82 (0.24)
	Identification score on appropriate vignettes (max = 1)	0.78 (0.28)
	Identification score on inappropriate vignettes (max = 1)	0.78 (0.19)
	Justification score on appropriate vignettes (max = 7)	2.01 (1.38)
	Justification score on inappropriate vignettes (max = 7)	2.04 (1.18)
Social (mal)adjustment	EASE total (max = 98)	57.9 (17.18)
	EASE ToM (max = 52)	27.84 (9.34)
	EASE Social Skills (max = 46)	30.06 (8.36)
	SCBE – Externalizing problems	68.69 (17.97)
	SCBE – Internalizing problems	70.94 (15.54)
	SCBE – Social competence	108.31 (27.72)
	SCBE – General adjustment	247.93 (51.44)
	SCBE – Depressive-happy	34.96 (8.28)
	SCBE – Anxious-secure	31.63 (9.06)
	SCBE – Isolated-integrated	33.87 (8.49)
	SCBE – Dependent-autonomous	28.99 (8.66)
	SCBE – Angry-tolerant	27.19 (9.45)
	SCBE – Aggressive-controlled	31.02 (7.38)
	SCBE – Egoistic-prosocial	27.04 (8.77)
	SCBE – Resistant-cooperative	33.24 (9.26)
	CBCL Externalizing Behaviors	16.36 (9.71)
	CBCL Internalizing Behaviors	16.21 (8.54)

**IDs, intellectual disabilities; CA, chronological Age; GDA, Global Developmental Age; VDA, Verbal Developmental Age; ToM, Theory of Mind; EASE, Social Adjustment Scale for Children; SCBE, Social Competence and Behavior Evaluation; CBCL, Child Behavior Checklist.**

### Measures

#### Wechsler Preschool and Primary Scales (WPPSI-III; Wechsler, 2004)

Four subtests – “information,” “vocabulary,” “block design,” and “matrix reasoning” – of the well-known WPPSI-III were administered. The results indicated the children’s verbal and non-verbal cognitive functioning and global developmental age. This evaluation ensured that children displayed a preschool developmental age, in order to meet the criteria for inclusion.

#### ToM-Emotions Tasks (Thirion-Marissiaux and Nader-Grosbois, 2008b)

ToM-emotions is a computerized instrument (on Eprime) that assesses the comprehension of causes and consequences of emotions (namely, joy, sadness, anger, and fear). There are three tasks: (1) The first is a preliminary task evaluating facial expression recognition for the four emotions; (2) The second task assesses the comprehension of causes of emotions. A script describing a situation of joy, sadness, anger, or fear is presented to the children, who have to predict the protagonist’s emotion depending on the story. Concretely, the children need to identify the emotion and justify their response for each story. Emotion recognition receives a score of 1 and the score for coherent justification is 0.5, with a maximum score of 6 for this task. (3) The third task evaluates the comprehension of consequences of emotions by presenting four scripts in which the protagonist feels joy, anger, sadness and fear respectively. Children have to choose one of the three behavioral responses suggested, according to the protagonist’s emotion. These options illustrate a socially adjusted, maladjusted, or neutral behavior. The choice of the socially adjusted card gets a score of 1, whereas the maladjusted or neutral card receives a 0. The children then have to justify their choice. A coherent justification receives 0.5. The maximum score for this third task is 6. The entire ToM-emotions instrument is thus scored out of 12.

Validation of the original version was conducted on 80 children with and without IDs and matched for preschool developmental age. The recorded evaluations revealed a high level of inter-judge agreement (between 95 and 98%, with Cohen’s kappa between 0.89 and 0.92; Pearson correlation coefficient between 0.93 and 0.96), based on each item score as well as for each task and emotion. A factor analysis revealed two factors related to the causes and consequences subscales (Thirion-Marissiaux and Nader-Grosbois, 2008b). Analysis of the computerized measure showed the same factors and a Cronbach’s alpha of 0.57 as well as a very high test-retest stability for the two subscales (between 0.56 and 0.68). For the present study, the Cronbach’s alpha coefficients are 0.34 and 0.27 respectively.

#### ToM-Beliefs Tasks (Thirion-Marissiaux and Nader-Grosbois, 2008a)

The ToM-beliefs tasks instrument evaluates the understanding of beliefs through five popular tasks. (1) The deception skills task (Oswald and Ollendick, 1989) assesses the ability of the child to deceive an adult by hiding a little object in his or her hands. (2) A change of representation task (Flavell et al., 1981) asks the child to infer what the adult sees on a specific image. (3) The third task is the appearance-reality task (Flavell, 1986). The experimenter presents an object with an appearance that differs from its real function (e.g., a pencil that looks like a flower). The child has to distinguish appearance from reality. (4) During the fourth task, the unexpected content task (Perner et al., 1987), the experimenter shows a Smarties box filled with pencils to the child and asks, “What is inside the box?” After demonstrating the content, experimenter fills the box with the pencils again and asks the child, “What did you think was in the box before it was opened?” and “What will your mother think is in the box if she has not seen inside it?” (5) The last task is the change of location task (Wimmer and Perner, 1983), corresponding to the well-known “Max and the transfer of chocolate” task. Each task gets a score of 1, with a maximum score of 5.

This measure was validated with typically developing preschoolers and children with IDs (ages 6–15) with a preschool developmental age. Evaluations were recorded and the following analysis demonstrated a very high inter-judge agreement (between 99 and 100%; Cohen’s kappa between 0.98 and 0.99; Pearson correlation coefficient between 0.99 and 1). A test-retest session revealed no significant difference (Thirion-Marissiaux and Nader-Grosbois, 2008a). Cronbach’s alpha for the present sample is 0.62.

#### ToM-Task Battery – French Version (Hutchins et al., 2008a; Nader-Grosbois and Houssa, 2016)

This battery was created to assess affective and cognitive ToM. Mental states are evaluated by means of nine tasks: (1) emotion recognition; (2) perspective taking; (3) inference of desire-based emotion; (4) inference of perception-based belief; (5) inference of perception-based action; (6) false belief; (7) inference of belief- and reality-based emotion and second order emotion; (8) message-desire discrepancy; and (9) second-order false belief. Children are asked control (e.g., What does Brigitte want? Where did Anthony put his book?), prompt (e.g., Where is the book now?), and test (e.g., Where will Anthony search his book?) questions. Only test questions are scored. Each task is scored 1 except for the three following: emotion recognition is scored 4 (1 point for recognition of joy, sadness, fear and anger respectively); perspective taking is scored 2 (since the child has to take the perspective of two protagonists); and inference of belief- and reality-based emotion and second-order emotion are scored 2 (1 point for recognition of emotion and of second-order emotion respectively). The total is scored out of 15. Subscores can be calculated to obtain affective, cognitive, and mixed scores.

This measure was validated for children with autism-spectrum disorder (ages 4.5–12) and revealed good internal consistency (α = 0.91) and test-retest reliability, considering different time variation between two administrations (Hutchins et al., 2008b). Another validation of the French version was carried out on typically developing preschoolers. Analysis revealed good internal consistency (α = 0.75) and test-retest reliability (*r* = 0.87) (Nader-Grosbois and Houssa, 2016). The reliability of this measure for the present sample is also good (α = 0.68).

#### Problem-Solving Task (RES, Barisnikov et al., 2004)

The problem-solving task estimates how children identify and judge a protagonist’s social behavior as appropriate or not. Fourteen images illustrate fictitious social situations involving either appropriate (5) or inappropriate (9) behaviors. For each image, children are asked to judge whether the behavior is appropriate or not (judgment score), to identify target behavior by social cues (identification score), and to justify their judgment (justification score). A score of 1 or 2 points is given for correct identification and judgment respectively. The justification score is determined by the children’s responses to the consequence for the protagonist (descriptive level: 2 points), to social consciousness (intersubjective level: 5 points), or to social rules (conventional level: 7 points). This measure mobilizes SIP skills. It is possible to differentiate scores depending on the social behavior depicted in the images. Five vignettes illustrate appropriate behaviors, and nine others illustrate inappropriate actions. In this study, identification, judgment, and justification scores are analyzed from responses to the vignettes, providing six subscores for either appropriate or inappropriate behaviors. The maximum total score is 140: 28 for judgment, 14 for identification, and 98 for justification.

The validation revealed an inter-judge agreement of 98% on a sample of children with and without IDs (Hippolyte et al., 2010). For the present study, Cronbach’s alpha is 0.87.

#### Social Adjustment Scales for Children (EASE; Hughes et al., 1997)

This questionnaire measures adults’ perception of children’s social adjustment. Parents estimate how frequent particular behaviors occur in daily interactions (rarely, relatively frequently or usually). Half of the items measure adaptive social skills (e.g., politeness, discipline or civility), and the other half assess social behaviors related to ToM abilities (e.g., considering others’ emotions, desires or beliefs), providing two subscores: one for social skills (maximum 46) and the other for ToM (maximum 52).

The two subscales have a good internal consistency, with Cronbach’s alpha coefficients of 0.77 and 0.79 respectively (Hughes et al., 1997). Similarly, good reliability was obtained for the present sample, with Cronbach’s alpha coefficients of 0.78 and 0.83.

#### Social Competence and Behavior Evaluation Scale (SCBE; LaFrenière et al., 1992)

This questionnaire assesses children’s socio-affective profile through 80 items. Parents evaluate to what extent their children display each behavior, using a 6-point Likert scale, from “never” to “always.” The questionnaire provides a complete profile in eight socio-affective domains: angry-tolerant, anxious-secure, depressive-happy, isolated-integrated, dependent-autonomous, resistant-cooperative, egoistic-prosocial, and aggressive-controlled. Each dimension is evaluated on a continuum emphasizing the child’s weaknesses and strengths. Some dimensions are related to the affective domain (depressive-happy; angry-tolerant; anxious-secure), while others reflect interactions with peers (isolated-integrated; egoistic-prosocial; aggressive-controlled) or with adults (dependent-autonomous, resistant-cooperative). The sum of the scores in certain specific dimensions gives four global scales: externalizing problems, internalizing problems, social competence, and general adjustment. The externalizing scale clusters four of the eight dimensions (angry-tolerant, resistant-cooperative, egoistic-prosocial and aggressive-controlled), while the internalizing scale brings together the other four (anxious-secure, depressive-happy, isolated-integrated, dependent-autonomous). The social competence scale covers 40 positive statements and assesses behaviors related to affective maturity, flexibility, and adequate adjustment during social interactions with peers or adults. The general adjustment scale reflects a global score through all 80 statements of this measure. These scores can be converted into T-scores, allowing the results to be compared with standards varying according to participants’ gender and developmental age (more or less than 4 years), and making it possible to identify difficulties or strengths. For all the scales and dimensions, T-scores lower than 38 or above 68 reflect weaknesses or strengths in comparison to a representative sample.

For the French version, the eight subscales display Cronbach’s alpha coefficients of between 0.79 and 0.82 and for the present sample between 0.78 and 0.90.

#### Child Behavior Checklist (CBCL; Achenbach, 1991)

This well-known questionnaire of 79 items assesses parents’ perception of children’s behavioral and emotional problems. Parents indicate the frequency of children’s behaviors on a 3-point Likert scale, from “not at all” to “often.” This produces, among other things, two scores for the presence of either internalizing or externalizing behaviors. Four subscales, namely “anxious/depressed,” “emotionally reactive,” “withdrawn,” and “somatic complaints,” determine the internalizing behavior score (clinical cutoff > 17), whereas the “attention problems” and “aggressive behavior” subscales are integrated to provide the externalizing behavior score (clinical cutoff > 24). These scores provide information about the sample’s clinical profile and potential risk of behavioral problems.

Cronbach’s alpha for the different subscales is between 0.63 and 0.86. For the present study, Cronbach’s alpha was 0.85.

### Procedure

All the described measures were administered to children and parents during a period of 2 weeks. The researcher used direct measures to evaluate the children’s cognition, ToM and SIP skills. The evaluation took place in a quiet room at the child’s school, during two 45 min sessions. At the same time, the parents received questionnaires about their perceptions of their child’s social adjustment and social and behavioral competence, including internalizing or externalizing behaviors problems. Parents could choose either to fill in the questionnaires at home or to make an appointment to fill them in with the researcher’s help. This help was requested by 80% of the parents. Given their low socioeconomic status and education level, many items were difficult for them to understand. During interviews, they also preferred to discuss the challenging behaviors they faced daily.

## Results

### Participants’ Characteristics

Table 1 presents average scores and standard deviations for the sample’s demographic and individual characteristics including chronological and developmental age and scores in ToM, SIP, and social abilities. Details about ages and demographic data are given above in the description of the sample. The ToM and SIP measures give indications of strengths and weaknesses, but not standards. Regarding ToM and SIP tasks, children’s scores were not below average, except for cognitive and mixed ToM Task Battery. In terms of socio-affective profile, T-scores for the SCBE scales results indicated that the children in the present sample displayed neither specific weaknesses nor strengths. T-scores for the four global scales (externalizing problems, internalizing problems, social competence, and general adjustment) were 47 respectively. For specific dimensions, T-scores corresponded in order to 49, 47, 48, 42, 45, 49, 48, 48, and 44. No T-scores for global scales and specific dimensions (ranging from 42 to 49) were lower than the cutoff (38). This demonstrated that these children with IDs had competence corresponding to those of a representative sample matched for developmental age. However, by comparing qualitatively the eight dimensions with each other, it can be observed on the one hand that with adults children were more irritable or frustrated and less autonomous and cooperative. On the other hand, scores revealed that they were perceived as particularly happy and controlled in their interactions with peers. In terms of behavior problems, children were at higher risk of developing internalizing problems, given that their score (mean = 16) was at a borderline level (according to CBCL standards).

### Inter-Correlations Between Individuals’ Characteristics and Skills in Social Cognition

Table 2 presents intercorrelations between individuals’ characteristics and competence related to ToM and SIP within the present sample. It reveals that global and verbal developmental ages were linked in a statistically significant way with all ToM (*r* between 0.252 and 0.674; *p* < 0.05) and SIP abilities (*r* between 0.348 and 0.683; *p* ≤ 0.001), whereas chronological age was related positively and significantly only to certain aspects, namely the total (*r* = 0.241; *p* < 0.05) and affective (*r* = 0.244, *p* < 0.05) scores of ToM Task Battery, ToM beliefs (*r* = 0.362; *p* ≤ 0.001), the judgment score on appropriate vignettes (*r* = 0.364; *p* < 0.05), the identification score on inappropriate vignettes (*r* = 0.228; *p* < 0.05), and the justification score on both appropriate (*r* = 0.276; *p* < 0.05) and inappropriate (*r* = 0.232; *p* < 0.05) vignettes. ToM and SIP abilities were highly and positively interrelated in a statistically significant way (*r* between 0.228 and 0.595; *p* ≤ 0.001).

**TABLE 2:** Spearman correlations between individuals’ characteristics, Theory of Mind and Social information processing skills.

		1	2	3	4	5	6	7	8	9	10	11	12	13	14	15	16	17	18
Individual	1. CA (in months)		0.319**	0.323**	0.086	0.241*	0.244*	0.137	0.213	0.118	0.081	0.169	0.362**	0.354*	0.185	0.360	0.228*	0.276*	0.232*
characteristics	2. GDA (in months)			0.915**	–0.287	0.570**	0.602**	0.256*	0.384**	0.483**	0.328**	0.481**	0.674**	0.501**	0.403**	0.507**	0.489**	0.579**	0.656**
	3. VDA (in months)				–0.237	0.566**	0.554**	0.252*	0.404**	0.580**	0.391**	0.582**	0.673**	0.476**	0.348**	0.493**	0.521**	0.592**	0.683**
	4. Family income					–0.137	–0.216	–0.054	–0.021	0.031	–0.121	0.052	–0.162	0.009	–0.147	–0.023	–0.141	0.095	–0.203
Explicit ToM measures	5. ToM Task Battery total (max = 15)						0.748**	0.820**	0.498**	0.344**	0.314**	0.245*	0.510**	0.507**	0.255*	0.499**	0.441**	0.495**	0.510**
	6. Affective ToM Task Battery (max = 6)							0.412**	0.319**	0.314**	0.369**	0.169	0.515**	0.397**	0.351**	0.399**	0.418**	0.392**	0.345**
	7. Cognitive ToM Task Battery (max = 6)								0.121	0.197	0.233*	0.153	0.224	0.356**	0.014	0.314**	0.242*	0.317**	0.272*
	8. Mixed ToM Task Battery (max = 3)									0.151	0.068	0.095	0.354**	0.377**	0.218	0.367**	0.182	0.268*	0.490**
	9. ToM emotions (max = 12)										0.716**	0.850**	0.496**	0.277*	0.259*	0.416**	0.507**	0.471**	0.401**
	10. ToM emotions – causes (max = 6)											0.392**	0.469**	0.213	0.228*	0.370**	0.426**	0.361**	0.302**
	11. ToM emotions – consequences (max = 6)												0.473**	0.327**	0.213	0.400**	0.545**	0.488**	0.412**
	12. ToM beliefs (max = 5)													0.505**	0.441**	0.563**	0.595**	0.562**	0.530**
Problem-solving task	13. Judgment score on appropriate vignettes (max = 2)														0.210	0.798**	0.484**	0.699**	0.503**
	14. Judgment score on inappropriate vignettes (max = 2)															0.292**	0.542**	0.147	0.407**
	15. Identification score on appropriate vignettes (max = 1)																0.737**	0.791**	0.597**
	16. Identification score on inappropriate vignettes (max = 1)																	0.656**	0.640**
	17. Justification score on appropriate vignettes (max = 7)																		0.669**
	18. Justification score on inappropriate vignettes (max = 7)																		

CA, Chronological Age; GDA, Global Developmental Age; VDA, Verbal Developmental Age; ToM, Theory of Mind; *p < 0.05; **p ≤ 0.001.

Table 3 presents correlations between individuals’ characteristics and abilities related to ToM and SIP on the one hand and their social adjustment competence and socio-affective profile on the other hand. The results indicate that global and verbal developmental ages are linked positively and significantly with social adjustment (*r* = 0.333 and 0.379 respectively; *p* ≤ 0.001). They are also linked in a statistically significant way with some dimensions of the socio-affective profile. Global developmental age is correlated with anxious-secure (*r* = 0.281; *p* < 0.05) and isolated-integrated (*r* = 0.272; *p* < 0.05) subscales while verbal developmental age is correlated with depressive-happy (*r* = 0.267; *p* < 0.05), anxious-secure (*r* = 0.378; *p* ≤ 0.001) and isolated-integrated (*r* = 0.357; *p* ≤ 0.001) subscales, whereas chronological age is not. Moreover, a higher verbal developmental age is associated in a statistically significant way with a lower risk of developing internalized problems (*r* = 0.273; *p* < 0.05) and with better social competence (*r* = 0.344; *p* ≤ 0.001). Affective ToM is associated positively and significantly with social adjustment (related to social skills; *r* = 0.234; *p* < 0.05) and competence (*r* = 0.375; *p* ≤ 0.001), as well as with some socio-affective dimensions (anxious-secure, *r* = 0.236; *p* < 0.05; isolated-integrated, *r* = 0.232; *p* < 0.05; dependent-autonomous, *r* = 0.266; *p* < 0.05). A good understanding of affective mental states is related in a statistically significant way to a lower risk of developing internalized problems (*r* = 0.242; *p* < 0.05) and externalizing behaviors (*r* = -0.267; *p* < 0.05). Cognitive ToM measured by ToM beliefs, is associated positively and significantly with social adjustment (*r* = 0.274; *p* < 0.05) and competence (*r* = 0.372; *p* ≤ 0.001). It is also correlated positively and significantly with anxious-secure (*r* = 0.256; *p* < 0.05) and dependent-autonomous (*r* = 0.259; *p* < 0.05) subscales of the SCBE measure.

**TABLE 3:** Spearman correlations between individuals’ characteristics and abilities in Theory of Mind and social information processing and their social adjustment and socio-affective profile.

		EASE			SCBE												CBCL	
		Total	ToM	Social Skills	Ext. prob.	Int. prob.	Social Compet.	General adjustment	Depressive-Happy	Anxious-Secure	Isolated-Integrated.	Dependent-Autonomous.	Angry- Tolerant	Aggressive-Controlled	Egoistic-Prosocial	Resistant-Coop.	EB	IB
Individual characteristics	CA (in months)	0.141	0.185	0.104	–0.087	–0.213	–0.010	–0.058	–0.183	–0.187	–0.162	–0.088	–0.084	–0.073	0.045	–0.103	–0.066	–0.121
	GDA (in months)	0.333**	0.349**	0.332**	–0.170	0.206	0.281−	0.194	0.210	0.281*	0.272*	0.221	–0.039	–0.144	–0.060	0.055	–0.067	–0.212
	VDA (in months)	0.379**	0.400**	0.378**	–0.086	0.273*	0.344**	0.275*	0.267*	0.378**	0.357**	0.227	0.038	–0.052	0.028	0.100	–0.043	–0.232
	Family income	0.057	0.010	0.098	0.099	–0.027	–0.087	–0.034	0.036	–0.228	–0.166	–0.035	0.106	0.167	0.016	0.077	–0.228	–0.184
Explicit ToM measures	ToM Task Battery total (max = 15)	0.222	0.219	0.235*	–0.014	0.243*	0.308**	0.266*	0.145	0.263*	0.277*	0.238*	0.067	0.065	0.082	0.126	–0.223	–0.236
	Affective ToM Task Battery (max = 6)	0.222	0.189	0.234*	0.082	0.242*	0.375**	0.321**	0.166	0.236*	0.232*	0.266*	0.083	0.152	0.207	0.221	−0.267*	–0.249
	Cognitive ToM Task Battery (max = 6)	0.102	0.094	0.117	0.052	0.165	0.159	0.158	0.066	0.143	0.134	0.110	0.095	0.127	0.105	0.055	–0.220	–0.198
	Mixed ToM Task Battery (max = 3)	0.149	0.188	0.125	–0.166	0.106	0.139	0.076	0.091	0.111	0.114	0.159	–0.064	–0.107	–0.155	0.038	–0.066	–0.202
	ToM emotions (max = 12)	0.412**	0.439**	0.369**	0.078	0.215	0.374**	0.330**	0.201	0.222	0.310**	0.191	0.195	0.131	0.278*	0.267*	0.086	–0.046
	ToM emotions – causes (max = 6)	0.341**	0.380**	0.280*	0.171	0.258*	0.451**	0.386**	0.176	0.261*	0.223	0.355**	0.270*	0.253*	0.338**	0.304**	0.105	0.052
	ToM emotions – consequences (max = 6)	0.276*	0.280*	0.271*	0.087	0.091	0.305**	0.275*	0.139	0.100	0.242*	0.033	0.214	0.186	0.289*	0.254*	0.029	–0.060
	ToM beliefs (max = 5)	0.274*	0.281*	0.275*	0.001	0.177	0.372**	0.297*	0.163	0.256*	0.225	0.259*	0.113	0.082	0.140	0.164	–0.186	–0.185
Problem-solving task	Judgment score on appropriate vignettes (max = 2)	0.191	0.190	0.214	−0.240*	–0.030	0.132	0.026	–0.019	0.046	0.013	0.066	–0.136	–0.020	–0.098	–0.053	–0.149	–0.140
	Judgment score on inappropriate vignettes (max = 2)	0.342**	0.308**	0.344**	–0.102	0.232*	0.256*	0.197	0.112	0.209	0.180	0.381**	0.057	–0.028	–0.042	0.054	0.071	0.019
	Identification score on appropriate vignettes (max = 1)	0.145	0.178	0.139	–0.087	0.040	0.229*	0.159	–0.016	0.077	0.046	0.174	0.082	0.077	0.083	0.021	–0.091	–0.039
	Identification score on inappropriate vignettes (max = 1)	0.205	0.229*	0.192	0.031	0.192	0.416**	0.341**	0.090	0.187	0.249*	0.330**	0.253*	0.215	0.212	0.110	–0.026	0.051
	Justification score on appropriate vignettes (max = 7)	0.244*	0.270*	0.241*	0.029	0.191	0.344**	0.297*	0.097	0.250*	0.160	0.232*	0.197	0.205	0.121	0.143	–0.180	–0.197
	Justification score on inappropriate vignettes (max = 7)	0.190	0.241*	0.171	–0.132	0.188	0.211	0.168	–0.038	0.236*	0.207	0.339**	0.048	–0.016	–0.083	0.001	–0.113	–0.226

CA, Chronological Age; GDA, Global Developmental Age; VDA, Verbal Developmental Age; ToM, Theory of Mind; EASE, Social Adjustment Scale for Children; SCBE, Social Competence and Behavior Evaluation; CBCL, Child Behavior Checklist; Ext prob., externalized problems; Int. prob., internalized problems; Compet., Competence; Coop., Cooperative; EB, Externalizing behaviors; IB, Internalizing behaviors. *p < 0.05; **p ≤ 0.001.

### Hierarchical Cluster Analysis With Reference to ToM and/or SIP Abilities

We applied a hierarchical agglomerative cluster analysis using Ward’s method and squared Euclidean distance in order to identify groups that presented different patterns in terms of ToM abilities, SIP competence, or both (using scores for ToM-emotions, ToM-beliefs and subscores for ToM task Battery and RES). The clustering allows exploration of the profile without an explanatory model.

**FIGURE 1:**
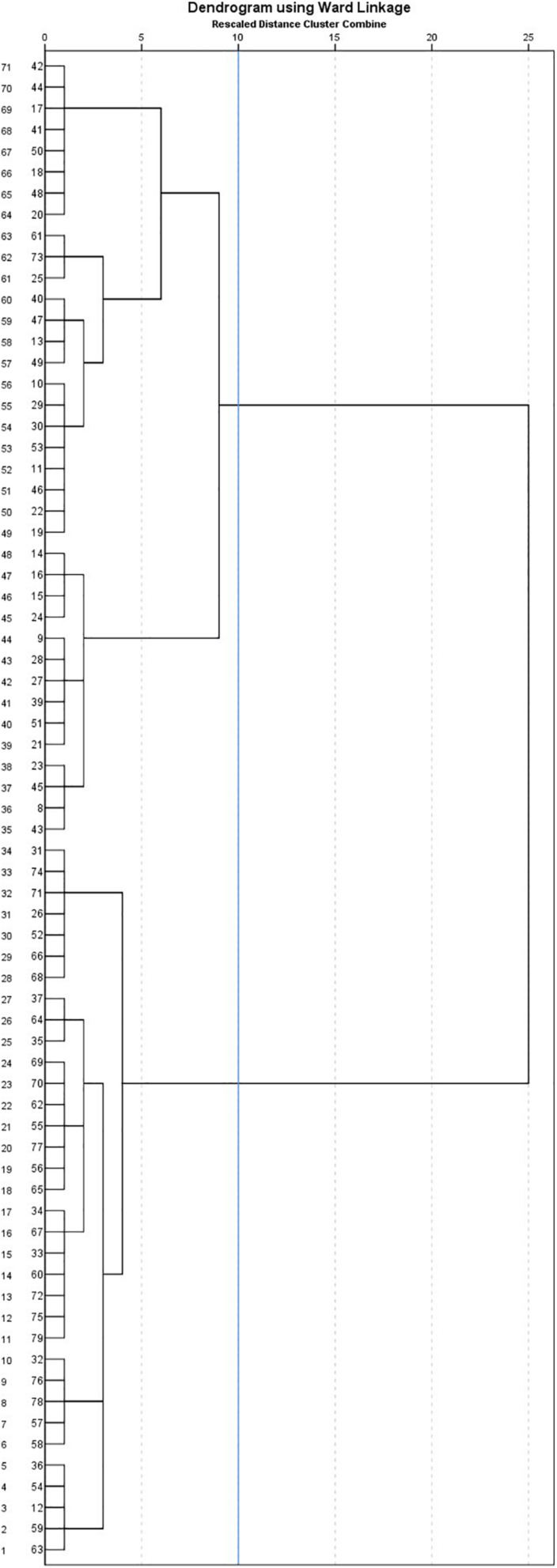
Dendrogram with added line indicating suggested stopping location, resulting from the application of hierarchical cluster analysis with Ward’s method and Euclidian distance and depending on Theory of Mind profile of children with intellectual disabilities.

The hierarchical cluster analysis depending on ToM profiles revealed two groups as shown in the dendrogram (see Figure 1). The average distance between these two clusters was 778.155.

Table 4 presents the individual characteristics and ToM competence of two clusters obtained through hierarchical cluster analysis using scores related to ToM task Battery, ToM-emotions and ToM-beliefs. Independent *t*-tests indicated some differences between these two clusters. Children in the second cluster had a higher chronological age [*t*(1) = 2.12; *p* = 0.037; *d* = 0.48] as well as a higher global [*t*(1) = 2.64; *p* = 0.011; *d* = 0.60] and verbal [*t*(1) = 3.13; *p* = 0.003; *d* = 0.71] developmental age, in comparison with children in the first cluster. In terms of ToM abilities, a marginal difference was revealed in the affective score for the ToM Task Battery [*t*(1) = 1.98; *p* = 0.051; *d* = 0.45], while significant differences were obtained in the understanding of consequence of emotions [ToM emotions-consequences; *t*(1) = 3.48; *p* = 0.001; *d* = 0.79] and of cognitive mental states [ToM beliefs; *t*(1) = 2.12; *p* = 0.034; *d* = 0.49]. The second cluster, displaying higher ages, also presented better ToM competence compared with the first cluster. Regarding social adjustment, a unique difference was significant. Children in the second cluster were perceived as more socially adjusted [EASE total; *t*(1) = 2.12; *p* = 0.026; *d* = 0.48], particularly in situations requiring the understanding of social conventions [EASE – social skills; *t*(1) = 2.12; *p* = 0.038; *d* = 0.48]. They also tended to be evaluated as more cooperative [*t*(1) = 1.74; *p* = 0.085; *d* = 0.40] when interacting with adults.

**TABLE 4:** Between-group comparisons of the two clusters obtained through hierarchical cluster analysis according to Theory of Mind abilities.

		Cluster 1 (*n* = 37)	Cluster 2 (*n* = 41)		
Variables		Mean (*SD*)	Mean (*SD*)	*X*^2^*^/^t* (1)	*d*
Sex (% boys)		67%	78%	1.08	
CA (in months)		104.62 (20.71)	114.59 (20.76)	2.12*	0.48
GDA (in months)		59.83 (13.83)	67.69 (12.49)	2.64*	0.60
VDA (in months)		58.03 (14.17)	67.26 (11.82)	3.13**	0.71
Family income		3.32 (0.98)	2.80 (1.15)	–1.49	
Explicit ToM measures	ToM Task Battery total (max = 15)	7.59 (2.31)	8.63 (2.33)	1.97^†^	0.45
	Affective ToM Task Battery (max = 6)	4.78 (1.22)	5.27 (0.95)	1.98^†^	0.45
	Cognitive ToM Task Battery (max = 6)	2.39 (1.14)	2.73 (1.66)	0.99	
	Mixed ToM Task Battery (max = 3)	0.53 (0.76)	0.71 (1.01)	0.82	
	ToM emotions (max = 12)	6.78 (2.35)	8.38 (2.06)	3.17**	0.72
	ToM emotions – causes (max = 6)	3.78 (1.59)	4.33 (1.29)	1.66	
	ToM emotions – consequences (max = 6)	3.53 (1.73)	4.85 (1.59)	3.48***	0.79
	ToM beliefs (max = 5)	2.57 (1.4)	3.21 (1.19)	2.16*	0.49
Social (mal)adjustment	EASE total (max = 98)	53.59 (17.04)	61.54 (15.99)	2.12*	0.48
	EASE ToM (max = 52)	25.78 (9.04)	29.66 (8.79)	1.91^†^	0.43
	EASE Social Skills (max = 46)	27.81 (8.58)	31.88 (8.34)	2.12*	0.48
	SCBE – Externalizing problems	67.81 (17.71)	69.51 (18.42)	0.40	
	SCBE – Internalizing problems	70.89 (14.71)	70.99 (16.48)	0.03	
	SCBE – Social competence	103.77 (25.81)	112.59 (29.11)	1.38	
	SCBE – General adjustment	242.48 (45.32)	253.11 (56.75)	0.89	
	SCBE – Depressive-happy	34.85 (8.09)	35.05 (8.57)	0.10	
	SCBE – Anxious-secure	31.56 (8.62)	31.69 (9.58)	0.07	
	SCBE – Isolated-integrated	32.35 (8.17)	35.31 (8.63)	1.52	
	SCBE – Dependent-autonomous	28.91 (9.54)	29.06 (7.86)	0.07	
	SCBE – Angry-tolerant	26.59 (9.05)	27.76 (9.91)	0.53	
	SCBE – Aggressive-controlled	30.76 (6.88)	31.26 (7.92)	0.29	
	SCBE – Egoistic-prosocial	26.10 (7.73)	27.92 (9.67)	0.89	
	SCBE – Resistant-cooperative	31.34 (8.59)	35.04 (9.62)	1.74^†^	0.40
	CBCL Externalizing Behaviors	17.09 (10.52)	14.41 (9.25)	1.05	
	CBCL Internalizing Behaviors	17.91 (9.38)	14.22 (8.74)	1.48	

CA, Chronological Age; GDA, Global Developmental Age; VDA, Verbal Developmental Age; ToM, Theory of Mind; EASE, Social Adjustment Scale for Children; SCBE, Social Competence and Behavior Evaluation; CBCL, Child Behavior Checklist; ^†^p ≤ 0.059; *p < 0.05; **p ≤ 0.01; ***p ≤ 0.001.

**FIGURE 2:**
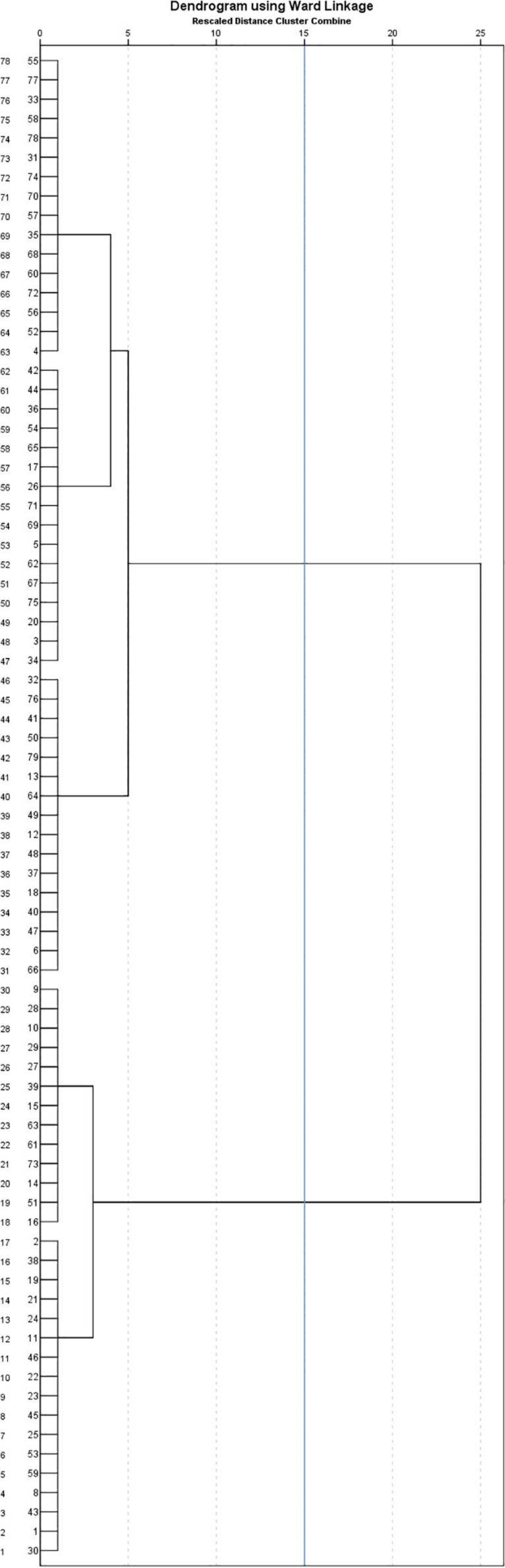
Dendrogram with added line indicating suggested stopping location, resulting from application of hierarchical cluster analysis with Ward’s method and Euclidian distance and depending on social information processing profile of children with intellectual disabilities.

The hierarchical cluster analysis depending on SIP profiles of the present sample revealed two clusters of cases, with an average distance between them of 113.070. The distribution of cases is presented in the dendrogram (see Figure 2).

In Table 5, independent *t*-tests demonstrated some differences between the two clusters obtained through hierarchical cluster analysis using RES scores. Compared with children in the first cluster, children in the second had a higher verbal developmental age [*t*(1) = 2.80; *p* = 0.008; *d* = 0.47] and better abilities at identifying [*t*(1) = 2.32; *p* = 0.028; *d* = 0.61] and justifying [*t*(1) = 2.35; *p* = 0.024; *d* = 0.58] socially inappropriate behavior. In terms of social adjustment, children in the second cluster were perceived as more socially competent [*t*(1) = 2.33; *p* = 0.036; *d* = 0.61] in comparison with those in the first cluster. They were perceived as more cooperative [*t*(1) = 2.20; *p* = 0.036; *d* = 0.59] in their interactions with adults and tended to be less aggressive [*t*(1) = 2.04; *p* = 0.052; *d* = 0.57] when interacting with peers.

**TABLE 5:** Between-group comparisons of the two clusters obtained through hierarchical cluster analysis according to Social information processing abilities.

		Cluster 1 (*n* = 56)	Cluster 2 (*n* = 22)		
Variables		*M* (*SD*)	*M* (*SD*)	*X*^2^*^/^t* (1)	*d*
Sex (% boys)		72%	73%	0.00	
CA (in months)		105.50 (20.13)	111.57 (21.54)	1.17	
GDA (in months)		59.45 (13.46)	65.74 (13.42)	1.86^†^	0.47
VDA (in months)		56.32 (12.81)	65.45 (13.29)	2.80**	0.47
Family income		3.23 (1.17)	3.10 (1.03)	–0.35	
Problem-solving task	Judgment score on appropriate vignettes (max = 2)	1.67 (0.46)	1.72 (0.51)	0.36	
	Judgment score on inappropriate vignettes (max = 2)	1.82 (0.24)	1.82 (0.23)	–0.01	
	Identification score on appropriate vignettes (max = 1)	0.74 (0.25)	0.79 (0.29)	0.71	
	Identification score on inappropriate vignettes (max = 1)	0.69 (0.23)	0.81 (0.16)	2.32*	0.61
	Justification score on appropriate vignettes (max = 7)	1.65 (1.42)	2.15 (1.36)	1.40	
	Justification score on inappropriate vignettes (max = 7)	1.57 (1.09)	2.23 (1.18)	2.35*	0.58
Social (mal)adjustment	EASE total (max = 98)	53.86 (17.68)	59.30 (16.45)	1.25	
	EASE ToM (max = 52)	25.14 (9.64)	28.87 (8.69)	1.58	
	EASE Social Skills (max = 46)	28.73 (8.57)	30.43 (8.71)	0.78	
	SCBE – Externalizing problems	63.52 (21.18)	70.59 (16.45)	1.35	
	SCBE – Internalizing problems	70.05 (14.55)	71.27 (16.01)	0.31	
	SCBE – Social competence	96.15 (27.58)	112.81 (26.64)	2.33*	0.61
	SCBE – General adjustment	229.72 (52.51)	254.68 (49.84)	1.84	
	SCBE – Depressive-happy	32.85 (8.89)	35.74 (7.99)	1.27	
	SCBE – Anxious-secure	30.05 (8.74)	32.22 (9.19)	0.94	
	SCBE – Isolated-integrated	31.72 (8.28)	34.67 (8.50)	1.35	
	SCBE – Dependent-autonomous	28.75 (8.57)	29.07 (8.77)	0.14	
	SCBE – Angry-tolerant	24.55 (10.61)	28.17 (8.89)	1.36	
	SCBE – Aggressive-controlled	27.80 (8.86)	32.21 (6.45)	2.04^†^	0.57
	SCBE – Egoistic-prosocial	24.77 (8.67)	27.87 (8.73)	1.36	
	SCBE – Resistant-cooperative	29.22 (9.86)	34.72 (8.66)	2.20*	0.59
	CBCL Externalizing Behaviors	17.35 (11.48)	15.30 (9.41)	–0.65	
	CBCL Internalizing Behaviors	14.88 (7.97)	17.03 (9.75)	0.86	

CA, Chronological Age; GDA, Global Developmental Age; VDA, Verbal Developmental Age; EASE, Social Adjustment Scale for Children; SCBE, Social Competence and Behavior Evaluation; CBCL, Child Behavior Checklist; ^†^p ≤ 0.06; *p < 0.05; **p ≤ 0.01; ***p ≤ 0.001; ****p = 0.000.

**FIGURE 3:**
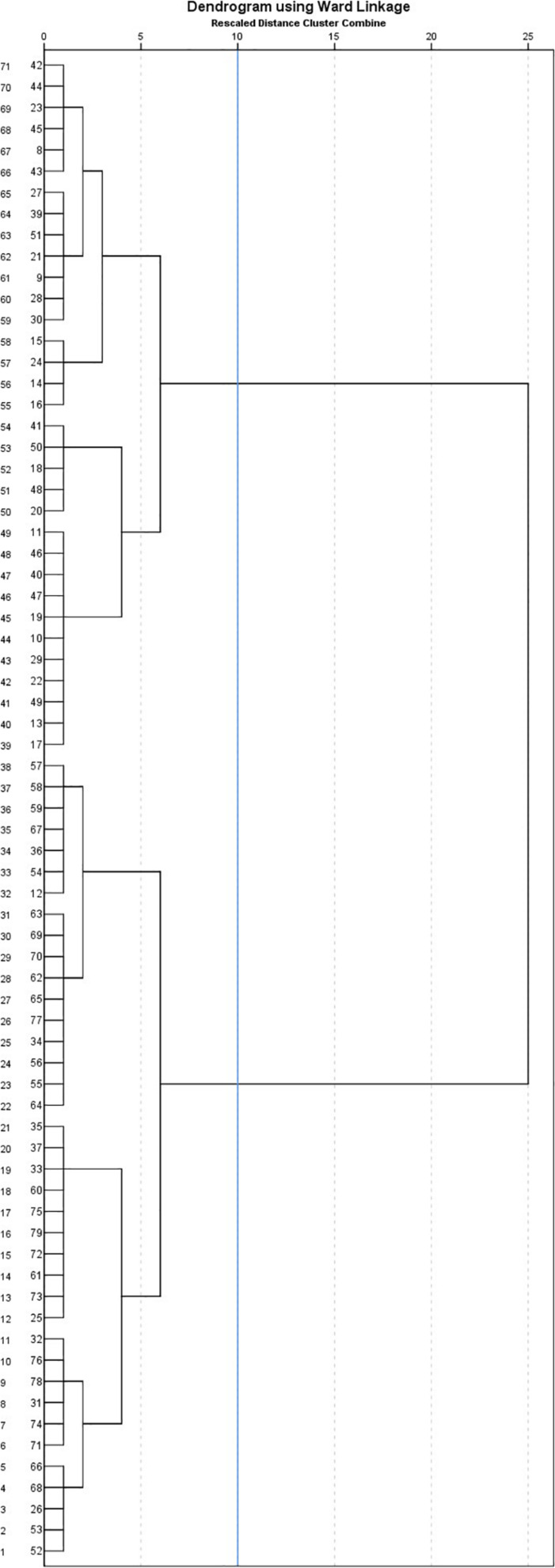
Dendrogram with added line indicating suggested stopping location, resulting from application of hierarchical cluster analysis with Ward’s method and Euclidian distance and depending on Theory of Mind and social information processing profiles of children with intellectual disabilities.

The hierarchical cluster analysis depending on ToM and SIP profiles indicated two clusters. The distribution of cases is presented in the dendrogram (see Figure 3). The average distance between the two clusters described below is 1036.334.

Table 6 presents the individual characteristics and socio-emotional competence of the two clusters obtained through hierarchical cluster analysis in terms of both ToM and SIP abilities. Independent *t*-tests highlighted differences between these two groups. In the first cluster, children displayed a lower developmental [*t*(1) = 4.16; *p* = 0.000; *d* = 0.94] and verbal [*t*(1) = 3.78; *p* = 0.000; *d* = 0.85] age, compared to children in the second cluster. Regarding ToM abilities, children in the second cluster presented more abilities in ToM than those in the first cluster. They displayed a better understanding of affective mental states [*t*(1) = 2.9; *p* = 0.005; *d* = 0.65], such as emotions [*t*(1) = 2.37; *p* = 0.021; *d* = 0.54] and of cognitive mental states [in cognitive ToM task Battery, *t*(1) = 2.49; *p* = 0.033; *d* = 0.58 and in ToM beliefs, *t*(1) = 2.18; *p* = 0.033; *d* = 0.49]. Similarly, compared with children in the first cluster, children in the second cluster identified [*t*(1) = 2.21; *p* = 0.030; *d* = 0.52] and justified [*t*(1) = 2.92; *p* = 0.005; *d* = 0.66] social behaviors in negative situations more easily. In terms of social adjustment, children with a lower developmental age and a lower level of social-cognitive skills also displayed fewer social competence [*t*(1) = -2.67; *p* = 0.009; *d* = 0.62] and adjustment [*t*(1) = -2.06; *p* = 0.043; *d* = 0.46]. These children were perceived as more anxious, especially in social groups, and as more isolated among peers. Children with a higher developmental age seemed to be more autonomous and cooperative with adults, in comparison with the younger cluster. Parents of these children reported more behavioral disorders, and more specifically a higher level of internalizing problems [*t*(1) = -2.62; *p* = 0.011; *d* = 0.61]. The CBCL indicated a mean corresponding to clinical level (>19) for this first cluster.

**TABLE 6:** Between-group comparisons of the two clusters obtained through hierarchical cluster analysis according to Theory of Mind and social information processing abilities.

		Cluster 1 (*n* = 36)	Cluster 2 (*n* = 42)		
Variables		Mean (*SD*)	Mean (*SD*)	*X*^2^*^/^t*	*d*
Sex (% boys)		64%	80%	2.87	
CA (in months)		105.97 (22.61)	113.19 (19.57)	–1.49	
GDA (in months)		57.66 (13.54)	69.37 (11.34)	−4.16****	0.94
VDA (in months)		57.01 (13.63)	67.9 (11.77)	−3.78****	0.85
Family income		3.39 (0.78)	2.85 (1.27)	1.71	
Explicit ToM measures	ToM Task Battery total (max = 15)	7.19 (2.3)	8.95 (2.13)	−3.48***	0.79
	Affective ToM Task Battery (max = 6)	4.66 (1.21)	5.36 (0.91)	−2.9***	0.65
	Cognitive ToM Task Battery (max = 6)	2.13 (1.26)	2.93 (1.5)	−2.49*	0.58
	Mixed ToM Task Battery (max = 3)	0.47 (0.76)	0.76 (0.99)	–1.35	
	ToM emotions (max = 12)	6.96 (2.46)	8.19 (2.07)	−2.37*	0.54
	ToM emotions – causes (max = 6)	3.9 (1.61)	4.21 (1.32)	–0.94	
	ToM emotions – consequences (max = 6)	3.69 (1.69)	4.65 (1.75)	−2.46*	0.56
	ToM beliefs (max = 5)	2.56 (1.39)	3.2 (1.19)	−2.18*	0.49
Problem-solving task	Judgment score on appropriate vignettes (max = 2)	1.69 (0.48)	1.71 (0.51)	–0.17	
	Judgment score on inappropriate vignettes (max = 2)	1.76 (0.28)	1.86 (0.18)	−1.88^†^	0.42
	Identification score on appropriate vignettes (max = 1)	0.77 (0.27)	0.79 (0.29)	–0.21	
	Identification score on inappropriate vignettes (max = 1)	0.72 (0.22)	0.82 (0.16)	−2.21*	0.52
	Justification score on appropriate vignettes (max = 7)	1.69 (1.44)	2.28 (1.28)	−1.88^†^	0.43
	Justification score on inappropriate vignettes (max = 7)	1.64 (1.12)	2.39 (1.14)	−2.92***	0.66
Social (mal)adjustment	EASE total (max = 98)	53.61 (17.33)	61.33 (15.8)	−2.06*	0.46
	EASE ToM (max = 52)	25.67 (9.01)	29.67 (8.81)	−1.98^†^	0.45
	EASE Social Skills (max = 46)	27.94 (8.86)	31.67 (8.18)	−1.93^†^	0.44
	SCBE – Externalizing problems	65.94 (16.91)	71.02 (18.8)	–1.22	
	SCBE – Internalizing problems	66 (15.98)	75.14 (14.02)	−2.62*	0.61
	SCBE – Social competence	99.32 (26.79)	115.94 (26.48)	−2.67**	0.62
	SCBE – General adjustment	231.25 (48.99)	262.11 (49.73)	−2.68**	0.62
	SCBE – Depressive-happy	33.17 (8.25)	36.48 (8.10)	–1.73	
	SCBE – Anxious-secure	29.18 (8.30)	33.71 (9.25)	−2.22*	0.51
	SCBE – Isolated-integrated	30.93 (8.81)	36.37 (7.43)	−2.84*	0.67
	SCBE – Dependent-autonomous	26.70 (9.92)	30.93 (6.97)	−2.09*	0.49
	SCBE – Angry-tolerant	25.16 (8.17)	28.92 (10.21)	–1.76	
	SCBE – Aggressive-controlled	30.25 (7.3)	31.67 (7.48)	–0.83	
	SCBE – Egoistic-prosocial	25.71 (7.06)	28.16 (9.94)	–1.23	
	SCBE – Resistant-cooperative	30.15 (9.28)	35.86 (8.51)	−2.74*	0.64
	CBCL Externalizing Behaviors	18.14 (10.62)	13.91 (9.09)	1.65	
	CBCL Internalizing Behaviors	19.04 (9.35)	13.79 (8.48)	2.18*	0.59

CA, Chronological Age; GDA, Global Developmental Age; ToM, Theory of Mind; EASE, Social Adjustment Scale for Children; SCBE, Social Competence and Behavior Evaluation; CBCL, Child Behavior Checklist; ^†^p ≤ 0.062; *p < 0.05; **p < 0.01; ***p ≤ 0.001; ****p = 0.000.

### Exploratory Factor Analysis

We applied an exploratory factor analysis in principal axis factoring with oblimin rotation on the subscores for ToM and SIP (see Table 7 for the loadings of each task on the factor and the percentage of explained variance). The first factor included affective ToM and social problem-solving skills related to inappropriate social behaviors. The second integrated cognitive ToM and social problem-solving skills related to appropriate social behaviors. The third one encompassed ToM competence linked to understanding of mixed mental states.

**TABLE 7:** Exploratory factor analysis in principal axis factoring with oblimin rotation in Theory of Mind and social information processing.

Factor	Loadings on factor
**SIPin-ToMAffCo**	
Judgment score of inappropriate vignettes	0.768
Identification score of inappropriate vignettes	0.740
Justification score of inappropriate vignettes	0.354
Affective ToM Task Battery	0.694
ToM-emotions	0.618
ToM-beliefs	0.690
**Percentage of explained variance**	**46.95%**
**Cumulative percentage**	**46.95%**
**SIPa-ToMCo**	
Judgment score of appropriate vignettes	–0.930
Identification score of appropriate vignettes	–0.879
Justification score of appropriate vignettes	–0.703
Cognitive ToM Task Battery	–0.332
**Percentage of explained variance**	**9.05%**
**Cumulative percentage**	**56%**
**ToM-mixed**	
Mixed ToM Task Battery	0.715
**Percentage of explained variance**	**4.86%**
**Cumulative percentage**	**60.86%**

**IP, social information processing; ToM, Theory of Mind.* The bold values corresponded to the percentage of explained variance and the cumulative percentage for each factor.*

## Discussion

The present study aimed to explore the social cognitive profiles of children with non-specific IDs. To do so, we investigated whether different clusters could be distinguished within one sample according to ToM and/or SIP competence and how these profiles of abilities were related to one another in children with IDs. Results indicated that children with IDs could be distinguished by their social cognitive profiles. Children who displayed better social cognitive abilities had higher chronological and/or global and verbal developmental ages, as well as better social, emotional, and behavioral competence and adjustment. The exploratory factor analysis revealed that ToM abilities and SIP competence are both used during positive or negative social interactions.

When hierarchical cluster analyses were used with respect to ToM abilities, SIP competence, or both, two groups were always identified, and some differences between them were revealed by comparing means, using independent *t*-tests. The two clusters obtained based on ToM abilities differed by chronological age as well as global and verbal developmental age. Children in the first cluster were younger and displayed lower ToM abilities, particularly in the understanding of emotions, consequences, and cognitive mental states. These results are in line with the literature, which has identified a positive and predictive relationship between developmental age and ToM abilities, notably in typically developing children (Grazzani et al., 2018; Conte et al., 2019) and in children with IDs (e.g., Charman and Campbell, 2002; Baurain and Nader-Grosbois, 2013; Nader-Grosbois et al., 2013). Children have to display a certain level of cognitive skills to understand mental states (Cicchetti et al., 1995). The difference in chronological age highlights the potential impact of social life experiences that become more diversified over time. As they get older, children experience more social interactions with different people, and this gives them opportunities to develop ToM abilities (Vygotsky, 1978). This conclusion also explains why the older children in this study score more highly on the ToM-beliefs measure, as they had first-hand experience of deception and of perspective taking. Compared with older children, the first cluster presented lower competence in social adjustment, particularly when they had to use and respect social conventions and rules. They also seemed to be slightly less cooperative with adults. Clustering based on SIP competence indicated two groups that differed by verbal developmental age. Studies have shown developmental delay in social interaction abilities (Guralnick, 2006) and deficits in receptive and expressive language in children with IDs (Sigafoos, 2000). These delays and deficits limit discussion about critical social situations or the use of language in order to guide the SIP process. Children who displayed a higher verbal developmental age found it easier to identify a social behavior as inappropriate and to justify it by considering the relationship between the protagonists or by referring to social rules. In typically developing children, the relationship between abilities to construct and decide to enact positive social behaviors and expressive (Ziv, 2013) as well as receptive (Conte et al., 2018) language has already been revealed. Children with higher SIP skills seemed to be more socially competent and, in particular, less aggressive and more cooperative. These two dimensions are related to the quality of social interactions with peers and with adults respectively. Based on these results, we could speculate that children with higher language and SIP skills could be perceived as more socially competent and therefore interact with others in a more appropriate way, leading to a virtuous circle. In the two clusters obtained on the basis of ToM and SIP skills, children differed from one another in verbal and global developmental age. The older group displayed better competence in affective and cognitive ToM as well as in SIP specifically related to negative situations. With respect to understanding emotions, the difference between the two groups was only in the comprehension of the consequences of emotions. Children in the first cluster displayed lower competence in social cognition and also presented a lower developmental age and lower social adjustment skills. Concretely, these children had a developmental age of 4 years and 9 months, in comparison with the children in the other group, who presented a developmental age of 5 years and 9 months. This observation is in line with previous studies reporting a predictive link between developmental age and ToM (Charman and Campbell, 2002; Abbeduto and Murphy, 2004; Thirion-Marissiaux and Nader-Grosbois, 2008c; Alevriadou and Giaouri, 2011; Nader-Grosbois et al., 2013) and a relationship between developmental age and social problem-solving abilities (Baurain and Nader-Grosbois, 2013; Nader-Grosbois et al., 2013) in children with IDs. It is also consistent with the empirical observation that typically developing children acquire competence in social cognition as their verbal and non-verbal cognitive capacities increase (Wellman and Liu, 2004; Schultz et al., 2010; Grazzani et al., 2018; Conte et al., 2019). It highlights the importance of considering children’s developmental age during assessment or intervention rather than their chronological age in order to take account of the proximal zone of development. Compared with others, children with this lower level of competence in social cognition presented more social maladjustment. They were perceived as less socially adjusted in social situations requiring an understanding of mental states or social rules. Children in this first cluster also seemed to be less socially competent in various situations, displaying less positive, appropriate, flexible, and prosocial behaviors. Specifically, these children were perceived as more anxious. In their interactions with peers, they seemed more isolated, while when interacting with adults, they tended to be less autonomous and cooperative. Social maladjustment has been generally associated with a deficit in social cognition, notably in ToM (Charman and Campbell, 1996; Jervis and Baker, 2004; Nader-Grosbois et al., 2013) and SIP (Baurain and Nader-Grosbois, 2012). Children in the first cluster also tended to present more internalizing problems, even at a clinical level: a number of studies have observed the presence of behavioral problems in children with IDs (Dekker et al., 2002; Baker et al., 2003; Dekker and Koot, 2003; Emerson, 2003; Nader-Grosbois et al., 2013; Bailey et al., 2019), notably internalizing problems (Merrell and Holland, 1997; Guralnick, 1999). Some studies have also reported a link between internalizing problems and a deficit in social cognition (van Nieuwenhuijzen et al., 2004; Thirion-Marissiaux and Nader-Grosbois, 2008c).

Hierarchical cluster analyses led to classifying cases into groups that differ from each other but also that include individuals who present common specific characteristics in each group (Yim and Ramdeen, 2015). With respect to differences, average distances between clusters indicated that when cluster analyses of ToM abilities were run, the two groups were more distant than the two clusters obtained according to SIP skills. These findings indicated that children with IDs differ more according to their ToM abilities than to their SIP skills. While differences between clusters were highlighted above, some similarity could also be underlined. In the three cluster analyses, no difference between the two clusters was obtained for gender, family income, or the presence of externalizing problems. It revealed a homogeneity in these variables in the present sample. When children are clustered in two groups depending on their ToM abilities, differences appeared in social adjustment but not in their socio-affective and behavioral profiles, whereas when children are clustered in two groups depending on their SIP skills, no difference was observed in social adjustment. The goal of the present analyses is not to reveal whether ToM and SIP abilities were related to social competence and adjustment, especially since significant correlations are obtained in preliminary analysis (see Table 3) between these variables. Instead, cluster analysis aimed to investigate how diverse observations could be grouped according to different characteristics or variables, as in the present study, social cognitive abilities.

While ToM and SIP are two distinct and specific concepts, they could have an influence on each other. The present results demonstrated a relationship between the ability to understand mental states and social problem-solving skills in children with IDs. In their model, Crick and Dodge (1994) underline the particular function of some mental states (namely intentions, emotions and thoughts) in selecting and enacting prosocial behavior. Mazza et al. (2017) even consider ToM competence as prerequisites for SIP. In their view, children first have to understand their own and other people’s mental states before processing social information in order to behave in a socially appropriate way. Similarly, Lemerise and Arsenio (2000), Lemerise et al. (2005) emphasize the role of emotions in SIP. In our results, affective ToM is related to problem solving during negative situations while cognitive ToM is linked to SIP when an individual faces appropriate social behavior. It seems that when children face hostile intentions, provocation, frustration, or rejection, they make more use of skills related to affective ToM, i.e., the understanding of emotions, whereas in helping or sharing situations they tend to use cognitive ToM. In typically developing children, Conte et al. (2018) highlight relations on one hand between emotion knowledge and prosocial behavior of helping, and on the other hand, between cognitive ToM and sharing. Given the present result, future research could investigate similar links in children with IDs. Concerning the first factor of the exploratory factor analysis (SIPin-ToMAffCo), it includes items that differentiate children with IDs particularly with different levels of social cognitive skills. In fact, in the present study, affective and cognitive ToM as well as SIP competence in negative situations are competence that clusters children into two different groups. It seemed that children have to consider other people’s perspective or intentions to display prosocial behavior. To go further, it would be interesting to study the effect of ToM on SIP. Mazza et al. (2017) investigate the role of ToM components on SIP leading to prosocial behaviors in a sample of children with autistic spectrum disorder. In their study, Mazza et al. (2017) reveal that in children with autistic spectrum disorder, ToM competence does not reduce social problem-solving difficulties, whereas the understanding of emotions and beliefs does help typically developing children to interpret social cues during SIP.

The present findings revealed an underlying structure between ToM and SIP skills in children with IDs. Results demonstrated that ToM and SIP profiles in children with IDs could be distinguished. Thanks to cluster analyses, differences and similarities were observed. It stresses the importance of considering social cognitive variables separately and together, as well as the weaknesses and the strengths when exploring a child with IDs profile. Moreover, professionals have to pay attention to the relation between ToM abilities and SIP competence in positive or negative social situations during assessment and intervention processes toward children with IDs.

### Future Perspectives

Some limitations should be taken into account when considering the present results. Yet, they provide insights into future research opportunities. The sample included children with non-specific IDs. Similar studies need to be conducted with children with distinct genetic syndromes (e.g., Down syndrome, Williams’s syndrome) and with a control group of typically developing children. These designs would lead researchers to explore whether the same factors and groupings apply to other samples. This research fit a clinical special education approach. Therefore, we focused on the underlying strengths and weaknesses and chose measures based on developmental age rather than the intelligence quotients of children with IDs. Future research could replicate a study with similar objectives and hierarchical cluster analyses adding children’s intelligence quotients. As for instruments, ToM-emotions tasks presented a low reliability score for the present sample. Even if they provided more information than facial emotion recognition, notably about the understanding of causes and consequences of emotions, related results have to be considered carefully. Nevertheless, the affective subscore of the ToM Task Battery could be used to gather information on the understanding of emotions and desires. Future research could create and validate an assessment device with animated virtual support featuring characters who undergo negative and positive social situations and express various emotions (as used in the Emotion trainer program, conceived by Silver and Oakes, 2001). This type of device should be validated with children with IDs and typically developing children, in order to evaluate emotions recognition and understanding of causes and consequences of emotions, in a more dynamic way. To collect information on ToM abilities in diverse contexts, parents and/or teachers could fill in a questionnaire such as the Theory of Mind Inventory (Hutchins et al., 2012), on their perception of children’s ToM abilities. Variability in social cognition profiles could be investigated considering abilities in receptive and expressive language, in executive functions and empathy (Hippolyte et al., 2010). For instance, several authors have demonstrated that low levels of prosocial behaviors are associated with low empathy-related abilities in preschoolers (Strayer and Roberts, 2004; Williams et al., 2014), and that social problem solving is stronger in more empathetic children and adolescents (Coie and Dodge, 1998; de Wied et al., 2007). It would therefore be interesting to explore the empathy profile of these children. Given the specific emotion-related socialization behaviors of parents of children with IDs (Rodas et al., 2016; Jacobs et al., 2019b; Légaré et al., 2019), it would be interesting to consider family environment in this kind of study: parental socialization of emotions (e.g., reactions and conversations) has been recently found to affect social adjustment (Jacobs et al., 2019b), emotion regulation, and ToM abilities (Jacobs et al., 2019a). To investigate more precisely the causal contribution of ToM and SIP to each other and to the social and emotional competence of children with IDs, longitudinal studies or experimental studies implementing ToM or SIP training need to be conducted. ToM and SIP training studies including a control group and pre- and post-test measures of social cognition, emotion regulation and social adjustment have already highlighted some particular effects (Jacobs and Nader-Grosbois, 2020a, b).

### Psychoeducational Implications

As far as interventions are concerned, the results underscore the importance of considering the developmental age as well as the chronological age of children with IDs. Clinicians need to adapt materials and goals to ToM and SIP profiles (i.e., strengths and weaknesses) and to life experience. Since children display difficulties with both affective and cognitive mental states, it is crucial to assess all nine mental states and to support the understanding of all of them. Similarly, children with IDs tend to present higher difficulties during negative social situations, so SIP intervention should focus on both appropriate and inappropriate social behaviors. The relation between developmental verbal age and better SIP skills also highlights the importance of encouraging children to use self-verbalization. This could be fostered by experimenters in training by using repetitive and successive key questions adapted to SIP. Given the link between ToM and SIP, it could be hypothesized that interventions that aim to promote ToM abilities could impact SIP abilities and vice versa. Finally, both ToM and SIP interventions seem crucial to help children with IDs to be more socially adjusted and to have appropriate social interactions, in order to ultimately assist their integration and social inclusion.

## Data Availability Statement

The datasets presented in this article are not readily available because consent filled by participants ensured that the data will be used only for this research and not shared. Requests to access the datasets should be directed to nathalie.nader@uclouvain.be.

## Ethics Statement

The studies involving human participants were reviewed and approved by Ethics committee of the Psychological Sciences Research Institute. Written informed consent to participate in this study was provided by the participants’ legal guardian/next of kin.

## Author Contributions

EJ collected and analyzed the data and wrote the manuscript. PS helped to collect and analyzed the data. NN-G supervised all the study and helped to write the manuscript. All authors contributed to the article and approved the submitted version.

## Conflict of Interest

The authors declare that the research was conducted in the absence of any commercial or financial relationships that could be construed as a potential conflict of interest.
